# Distal-to-proximal progression of apophyseal injuries with age in male youth academy footballers: a two-season prospective cohort study of 16,024 player-seasons

**DOI:** 10.1136/bjsports-2024-109612

**Published:** 2025-10-05

**Authors:** Chelsea Oxendale, Matthew Green, Keith Stokes, Sean Cumming, Gemma Nicole Parry, Sean Williams

**Affiliations:** 1 University of Bath, Bath, UK; 2 Liverpool John Moores University, Liverpool, UK; 3 Elite Performance Manager, Football Association Premier League, London, UK

**Keywords:** Injuries, Football, Knee injuries, Sporting injuries

## Abstract

**Objectives:**

Youth football players are vulnerable to apophyseal injuries, which can have long-term consequences for health and performance. The objective was to assess the incidence, severity and burden of apophyseal injuries among U9–U21 academy football players over two seasons.

**Methods:**

Time-loss injuries and match/training exposure were tracked in male academy football players (U9–U21) from Premier League and Category One Academies. Apophyseal injuries were identified in a cohort of 16 024 player-seasons using Orchard Sports Injury Classification System (OSICS) codes, and their incidence, severity and burden were analysed.

**Results:**

A total of 10 589 injuries were reported, including 603 apophyseal injuries. These injuries followed a distal-to-proximal progression with age, occurring most frequently in the ankle/foot in younger players (U9, U10 and U11), followed by the knee (U12) and hip/groin in older players (U15, U16 and U18). Across all player phases (U9–U21), injury burden (days/1000 hours) was higher in the hip/groin (3.5) and knee (3.4), compared with the ankle/foot (2.2) and pelvis/sacrum (1.4), with the highest apophyseal incidence (~0.4 injuries/1000 hours) and burden (~20 days/1000 hours) observed in the U12–U16 group, regardless of injury location. A significant trend of increasing injury severity (median days) was observed with age: U9–U11 (20), U12–U14 (29), U15–U16 (38) and U18–U21 (35).

**Conclusions:**

Apophyseal injuries exhibit a distal-to-proximal progression with age, with the highest injury burden observed at the hip/groin and knee regions and in the U12–U16 group. These findings can help inform injury mitigation strategies adopted in youth academy football.

**WHAT IS ALREADY KNOWN ON THIS TOPIC**

- Apophyseal injuries account for a considerable portion of all musculoskeletal injuries in male academy football.
- The severity of apophyseal injuries varies based on their type and location, with hip and groin injuries being the most severe.

**WHAT THIS STUDY ADDS**

- This study reaffirms the distal-to-proximal progression of apophyseal injury location with age in a large academy cohort and highlights the increasing severity of these injuries with age.
- Across player phases, the hip/groin and knee regions had the highest injury burden, with hip/groin apophyseal injuries resulting in nearly twice the number of days missed compared with ankle/foot apophyseal injuries.
- Apophyseal injuries to the ankle/foot and knee carry the second-highest injury burden among all musculoskeletal injuries in the U9–U11 and U12–U14 groups, respectively.
- The burden of apophyseal injuries was highest in the U12–U16 groups and comparable between the U9–U11 and U18–U21 groups.

**HOW THIS STUDY MIGHT AFFECT RESEARCH, PRACTICE OR POLICY**

- This applied study provides comprehensive data on apophyseal injuries across all development phases in academy football, providing valuable insights to inform stakeholder education on the topic.
- The findings can guide the development of targeted strategies to mitigate growth-related injuries, emphasising the need for age and location-specific approaches implemented across all player development phases.

## Introduction

 The Elite Player Performance Plan (EPPP), introduced in 2012, is a long-term strategy to develop more talented homegrown players.^1^ In the 2021/22 season, around 14 200 youth football players were registered in academies across England and Wales,^1^ participating in both training and competitive matches. These youth players are required to perform numerous high-intensity movements, including rapid changes of direction, accelerations, decelerations, jumping, landing and football-specific actions.^2^ Despite improvements in the physical fitness of youth football players over the past decade,^3^ the sport poses an injury risk, with an overall incidence of six injuries per 1000 hours in youth football.^4 5^ Injury incidence tends to rise with chronological age as players mature from childhood to adulthood,^6^ with some injuries linked to the rapid and uneven growth phases during adolescence. During peak-height velocity, athletes are at a greater risk for bone and growth plate injuries,^7^ contributing to a higher overall injury burden in academy players,^8^ while muscle injuries are more common after the adolescent growth spurt.^6^

Apophyses are secondary bone growth centres serving as attachment sites for tendons or ligaments.^9^ These areas are particularly vulnerable to injury in developing youth athletes,^10 11^ typically caused by repetitive loading^12^ and exposure to forces that their still-maturing skeletal structures cannot handle. In youth football, apophyseal injuries account for 5–15% of all musculoskeletal injuries,^13^,^15^ with injuries at the ankle (Sever’s disease) and knee (Osgood-Schlatter disease) accounting for~14% of all musculoskeletal injuries sustained between the ages of 11 and 13.^15^ Notably, apophyseal injuries represent 30% of all severe injuries, defined as those causing more than 4 weeks of absence from matches or training,^14^ and can lead to an average of 55–60 days of time loss.^16 17^ These injuries can cause pain, weakness, disability and limitations in sports participation, significantly impacting both player development and long-term health.^11^

While some studies have explored the proportion and incidence of apophyseal injuries in youth football,^13 15 16 18^ few studies have examined the burden of apophyseal injuries,^14 17 19 20^ and these data are typically drawn from a single academy.^14 19 20^ Injury burden better reflects the impact of injuries compared with incidence or severity alone^21^ and has been negatively associated with player progression in academy football.^20^ The impact of apophyseal injuries therefore remains limited and warrants further investigation to aid the development of effective mitigation strategies. Few studies have also explored which apophyseal sites are most vulnerable, relative to player age. Specifically, while the mean age of 12 years for ankle apophyseal injuries^16^ aligns with the more frequent foot, ankle and knee apophyseal injuries in 10–12-year-olds,^13^ and the higher occurrence of hip apophyseal injuries in 12–14-year-olds,^13^ these findings are based on data from single academies. More comprehensive studies of apophyseal injuries in youth football are needed to establish consistent epidemiological data^11^ and guide the development and implementation of targeted injury mitigation strategies.^22^ Therefore, the aim of this study was to describe the incidence, severity, and burden of apophyseal injuries among U9–U21 male academy football players over two seasons, and to analyse the location and type of injuries relative to chronological age.

## Method

### Study design and population

This study utilised a prospective cohort study design, focusing on elite male youth academy football players from Premier League and Category One Academies, the highest tier of academy football in England and Wales. The study sample included 7927 players across 29 academies during the 2021/22 season and 8097 players across 31 academies in the 2022/23 season. Data were collected over two seasons, from July 2021 to June 2023, spanning 10 age groups (U9, U10, U11, U12, U13, U14, U15, U16, U18 and U21) and four player development phases: Foundation Development Phase for U9-U11, Early Youth Development Phase for U12-U14, Late Youth Development Phase for U15-U16 and Professional Development Phase for U18-U21.

Player injury data were routinely collected as part of the Premier League injury audit process, in accordance with the Premier League’s Player and Related Persons Privacy Policy, to which players and parents provide assent and consent during academy registration. The policy permits the sharing of data with academic institutions for research and analysis. Ethical approval for this study was granted by the University of Bath Research Ethics Approval Committee for Health (Ref: 5028–5186), allowing the use of de-identified data.

### Patient and public involvement

Football academy staff contributed to the study’s conception. Discussions at the Premier League Academy Injury and Illness Surveillance Project Steering Group identified the need for focused research on growth-related injuries. However, no players or parents were directly involved in the research.

### Equity, diversity and inclusion statement

The author group included two women and four men and consisted of junior, mid-career and senior researchers from different disciplines; however, all authors are from one country. Our study population included male athletes from different socioeconomic backgrounds participating in elite football academies; thus, findings may not be generalisable to female athletes or settings with fewer resources.

### Data collection

All musculoskeletal injuries sustained by academy players were recorded by a medical staff member at each academy using the Premier League’s online Performance Management Application (the Sports Office, UK). Each academy employed three full-time physiotherapists, all registered with the Health and Care Professions Council and holding an advanced trauma qualification, as a minimum requirement. A doctor, physiotherapist or sports therapist with a current advanced trauma qualification was present at all matches, while a physiotherapist or sports therapist with the same qualification was present at all training sessions. All academies operated within a centralised injury surveillance system, with clear guidance provided regarding injury definitions and classification procedures.

Injuries were recorded in a standardised format, following procedures for injury surveillance in football.^23^ Each injury was classified according to the Orchard Sports Injury Classification System (OSICS v10.1).^24^ Data collected included the date of injury, return date, injury location, type, cause and onset, as well as the event and activity during which the injury occurred (ie, match or training). For injuries without a recorded return date, an estimated date of return was used when players were still injured. Injuries were defined as time-loss injuries, adapted from the work of Fuller *et al*,^23^ in line with recent recommendations^25^:

‘Any physical complaint sustained by a player that results in the player being unable to take a full part in future normal training activities and/or match play for one or more days following the day of injury’*.*

Any injuries resulting in partial time loss were not included in the analysis. The definition of a recurrent injury was based on previous literature^23^ and aligned with the recent IOC consensus statement^25^:

‘An injury of the same type and at the same site as an index injury and which occurs after a player’s return to full participation from the index injury’.

An injury recurrence field was included and recorded as a binary variable (yes/no) to indicate whether an injury was classified as recurrent. Injury severity was defined as the number of days lost per injury event. The severity data were then aggregated across injury types and player groups to calculate median severity or average severity.^26^

In addition, match and training exposure time, measured in minutes for each academy, was recorded in the Player Management Application. The total match and training minutes were calculated for each age group and player phase by aggregating the summed player exposure for each match and training session (i.e., session duration x number of players in attendance), across all academies over the seasons. This exposure was converted into hours and subsequently used to determine injury incidence and burden. In cases where injuries occurred outside of a player’s chronological age group (e.g., a U12 player participating in a U13 match), the injury data were attributed to the player’s chronological age group to ensure consistency with their age-specific exposure data. All injury and exposure data were de-identified and exported by a third-party company and shared with the researchers. The total number of players registered for each academy was also exported to calculate injury prevalence. Four academies used a different electronic platform to record injuries, with their data anonymously provided directly to the researchers. Initially, the data were ‘cleaned’ using a custom-built script in R studio to standardise formatting. Researchers then verified the data by checking for compliance in injury and exposure fields, and any discrepancies were clarified through direct contact with the respective academies.

### Data analysis

Apophyseal injuries were identified using the OSICS code by selecting all injury codes that began with the prefix ‘JT’, corresponding to traction injuries and apophysitis/avulsion fractures, for further analysis. Each apophyseal injury was linked to a specific injury location in the exported dataset, categorised as ankle/foot, knee, hip/groin or pelvis/sacrum. Injuries involving the iliac crest, ischial tuberosity and pelvis were classified as pelvis/sacrum, while those involving the AIIS, ASIS and hip/groin region were classified as hip/groin. The cumulative prevalence of apophyseal injuries was calculated by dividing the number of apophyseal injuries by the total number of player-seasons. The proportion of total apophyseal injuries was also calculated, and a χ^2^ test of independence was conducted to examine the association between age group and injury location on injury counts. *Post hoc* standardised adjusted residuals greater than |1.96| were used to identify significant differences. Cumulative prevalence and the proportion of apophyseal injuries were presented relative to age group (e.g., U9), while all subsequent analyses were presented relative to player development phase (e.g., U9-U11).

Apophyseal injuries resulting in the highest injury burden were ranked based on the first two/three letters of the OSICS code and the total days lost. The incidence (injuries / 1000 hours), median severity and burden (days lost / 1000 hours) of match and training-related apophyseal injuries only were calculated relative to player developmental phase and lower limb body location.^25 27^ Incidence and burden values were reported with 95% CIs using the Poisson method,^28^ while the IQR was calculated for injury severity. Differences in incidence and burden were determined by non-overlapping 95% CIs. A Jonckheere-Terpstra test was used to assess statistical trends in severity across ordinal player phases, followed by *post hoc* pairwise multiple comparisons. The alpha level for statistical significance was set at 0.05, and a Bonferroni correction was applied to *post hoc* multiple comparisons, where appropriate. Analysis and reporting of results are consistent with procedures for injury surveillance in football^25^ and the CHecklist for statistical Assessment of Medical Papers.^29^

## Results

A total of 10 589 injuries, resulting in 3 79 476 days lost, were recorded over the 2021–22 and 2022–23 seasons, across a sample of 16 024 player-seasons. Of these, 603 were apophyseal injuries, representing 5.7% of all injuries and accounting for 26 789 days lost (7.1%). The median number of apophyseal injuries per academy per season was 8 (IQR 4–15), with a median total of 459 days lost (IQR 174–713 days). A total of 195 apophyseal injuries (32%) became symptomatic during match sessions and 262 (43%) became symptomatic during training sessions. An additional 146 apophyseal injuries became symptomatic during non-club-related activities (n=43, 7%), other club-related activities (n=12, 2%) or were reported with unspecified activity types (n=91, 15%). Data on recurrent injuries were available for 522 apophyseal injuries, of which 23 (4.4%) were reported as recurrent.

### All apophyseal injuries

**Figure 1:**
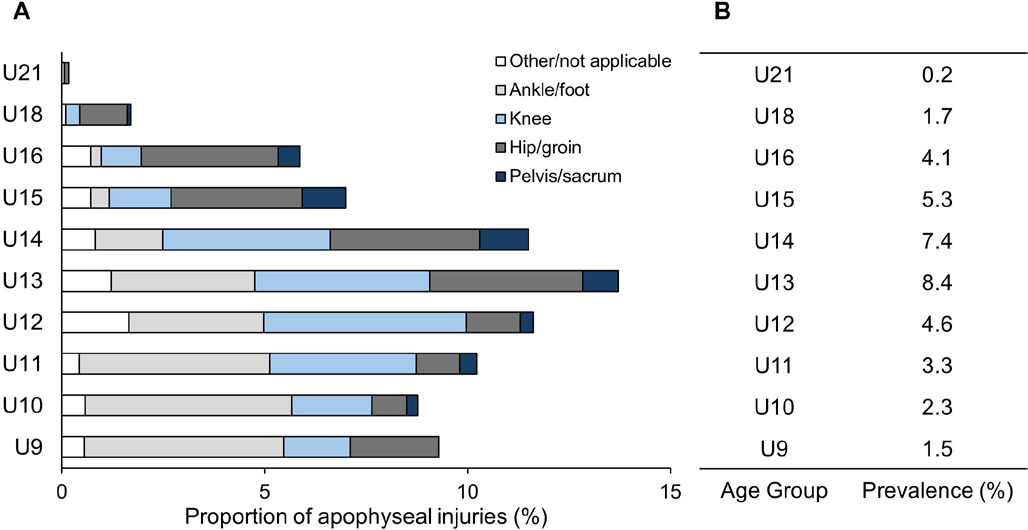
(A) The proportion of apophyseal injuries relative to total injuries for each age group during the 2021–22 and 2022–23 seasons is presented, along with the distribution of apophyseal injuries by location. (**B**) The cumulative prevalence (number of apophyseal injuries / number of player-seasons) of apophyseal injuries for each age group.

The proportion of apophyseal injuries, relative to total injuries, and the cumulative prevalence of apophyseal injuries across each age group are presented in figure 1. The overall cumulative prevalence of apophyseal injuries was 3.7%, with the U13 group demonstrating the highest cumulative prevalence (8.4%) and proportion of apophyseal injuries (13.6%). Of the 603 apophyseal injuries recorded, the distribution by body location was as follows: pelvis/sacrum 9%, hip/groin 31%, knee 30% and ankle/foot 21%, with 9% classified as other/not applicable.

A χ^2^ test of independence indicated a significant association between age and injury location (*X^2^(36)* = 163.21, p<0.001). Specifically, ankle/foot apophyseal injuries were significantly more frequent in the U9, U10 and U11 groups (SAR=3.2–5.1) and less common in the U14, U15, U16 and U18 groups (SAR=−2.1 to −3.5) than expected. Knee apophyseal injuries occurred more frequently than expected in U12 players (SAR=2.6) and less frequently in U16 players (SAR=−2.4). Hip/groin apophyseal injuries were less frequent in the U10, U11 and U12 groups (SAR=−2.8 to −4.0), but significantly more frequent in the U15, U16 and U18 groups (SAR=2.8–4.7). Additionally, pelvis/sacrum apophyseal injuries occurred more frequently in U15 players (SAR=2.7) than expected.

Apophyseal injuries accounted for the highest sum of days lost among all musculoskeletal injuries in the U9–U11 and U12–U14 age groups, ranking first and contributing 11% and 16% of total days lost, respectively, based on the first two letters of the OSICS code. In the U15–U16 and U18–U21 groups, apophyseal injuries ranked 3rd and 20th in the sum of days lost, accounting for 9% and 1% of total days lost, respectively. When analysed by anatomical location, the proportion of total days lost due to apophyseal injuries was distributed as follows: pelvis/sacrum 11%, hip/groin 31%, knee 29% and ankle/foot 18%.

A more detailed breakdown of apophyseal injury types, classified using the first three letters of the OSICS code, is provided in table 1. Ankle/foot and knee apophyseal injuries accounted for the second highest sum of days lost in the U9–U11 and U12–U14 groups, respectively, contributing to 5–6% of all days lost (table 1). In particular, Severs disease was the leading cause of apophyseal injury sum of days lost in the U9-U11 group, while Osgood-Schlatter disease had the highest sum of days lost in the U12–U14 group. For the U15–U16 and U18–U21 groups, apophyseal injuries with the highest sum of days lost occurred in the groin/hip area, specifically affecting the anterior superior iliac spine.

**Table 1:** The location and type of the top two apophyseal injuries with the highest sum of days lost are ranked relative to all musculoskeletal injuries for each player phase, based on the first three letters of the OSICS code

Player phase	Injury rank	Injury location and type	Number of injuries (% of total for phase)	Sum of days lost (% of total for phase)	Median time-loss (IQR)
FDP	2nd	**Injury to apophysis at ankle/foot**	**48 (5)**	**1589 (6)**	**28 (13–48)**
U9–U11		*Severs disease*	*48 (5)*	*1589 (6)*	*28 (13–48)*
Injury rank is out of 185	9th	**Injury to apophysis at knee**	**27 (3)**	**810 (3)**	**23 (13–39)**
		*Sinding-Larsen-Johansson syndrome*	*20 (2)*	*514 (2)*	*20 (13–35)*
		*Osgood-Schlatter disease*	*5 (<0.5)*	*182 (1)*	*23 (22–34)*
Early YDP	2nd	**Injury to apophysis at knee**	**114 (4)**	**4886 (5)**	**29 (17–65)**
U12–U14		*Osgood-Schlatter disease*	*79 (3)*	*3715 (4)*	*29 (13–72)*
		*Sinding-Larsen-Johansson syndrome*	*26 (1)*	*810 (1)*	*28 (20–49)*
Injury rank is out of 281	5th	**Injury to apophysis at groin/hip**	**80 (3)**	**3580 (4)**	**30 (20–51)**
		*Apophysitis/avulsion fracture AIIS*	*45 (2)*	*2060 (2)*	*29 (20–51)*
		*Apophysitis/avulsion fracture ASIS*	*16 (1)*	*673 (1)*	*33 (24–51)*
Late YDP	7th	**Injury to apophysis at groin/hip**	**72 (3)**	**3204 (4)**	**33 (18–58)**
U15–U16		*Apophysitis/avulsion fracture ASIS*	*30 (1)*	*1332 (2)*	*38 (23–73)*
		*Apophysitis/avulsion fracture AIIS*	*28 (1)*	*1140 (1)*	*23 (9–35)*
Injury rank is out of 280	11th	**Injury to apophysis at knee**	**28 (1)**	**1655 (2)**	**50 (27–88)**
		*Osgood-Schlatter disease*	*22 (1)*	*1312 (2)*	*49 (28–98)*
		*Sinding-Larsen-Johansson syndrome*	*6 (<0.5)*	*343 (<0.5)*	*66 (27–68)*
PDP	30th	**Injury to apophysis at groin/hip**	**25 (1)**	**1048 (1)**	**33 (24–58)**
U18–U21		*Apophysitis/avulsion fracture ASIS*	*14 (<0.5)*	*638 (<0.5)*	*35 (27–64)*
		*Apophysitis/avulsion fracture AIIS*	*3 (<0.5)*	*142 (<0.5)*	*71 (40–103)*
Injury rank is out of 344	52nd	**Injury to apophysis at knee**	**9 (<0.5)**	**517 (<0.5)**	**44 (19–73)**
		*Osgood-Schlatter disease*	*7 (<0.5)*	*486 (<0.5)*	*47 (32–80)*
		*Sinding-Larsen-Johansson syndrome*	*1 (<0.5)*	*29 (<0.5)*	*29 (N/a)*

In some cases, specific injury types were not provided, so totals per injury type may not sum to the overall number of injuries for that player phase.

FDP, Foundation Development Phase; OSCIS, Orchard Sports Injury Classification System; PDP, Professional Development Phase; YDP, Youth Development Phase.

### Match and training apophyseal injuries

Across all player phases (U9 – U21), the total number of match and training apophyseal injuries at the ankle/foot, knee, hip/groin and pelvis/sacrum was 94, 131, 144 and 43, respectively. Injury burden was higher in the hip/groin (3.5 days lost / 1000 hours; 95% CI 3.0 to 4.2) and knee (3.4 days lost / 1000 hours; 2.8–4.0) compared with the ankle/foot (2.2 days lost / 1000 hours; 1.8–2.7) and pelvis/sacrum (1.4 days lost / 1000 hours; 1.0–1.8).

**Figure 2:**
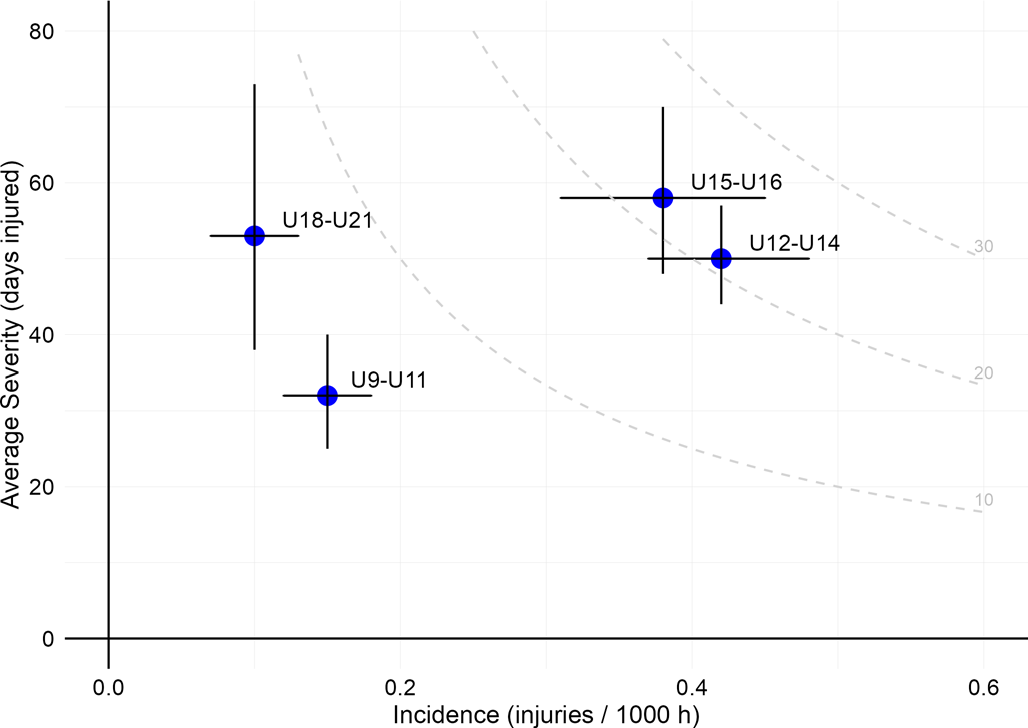
Risk matrix based on incidence and average severity (time-loss per injury event) for all apophyseal injuries, relative to player phases. Each blue point represents the injury burden, and the grey dashed lines represent points with an injury burden equal to 10, 20 and 30 days lost / 1000 hours. The vertical and horizontal error bars represent the 95% CI.

Injury incidence ranged from 0.10 to 0.42 across player phases, with higher rates in the U12–U14 and U15–U16 groups compared with the U9–U11 and U18–U21 groups (table 2 and figure 2). Injury burden was also higher in the U12–U14 (19.3 days lost / 1000 hours) and U15–U16 groups (20.3 days lost / 1000 hours) compared with U9–U11 and U18–U21. A significant trend for a higher severity with player phase was observed (*T_JT_*=33 006, *Z*=3.456, p<0.001). Specifically, severity for U12–U14 (*T*=8973, p=0.031) and U15–U16 (*T*=4797, p=0.001) was higher than U9–U11, while the difference between U9–U11 and U18–U21 approached significance (*T*=1549, p=0.057).

**Table 2:** Incidence, severity and burden of apophyseal match and training injuries relative to player phase and body location

Player phase and body location	Number of injuries	Injury incidence (injuries / 1000 hours) (95% CI)	Median time-loss (IQR)	Injury burden (days lost / 1000 hours) (95% CI)
**FDP** *(U9–U11)*	**74**	**0.15 (0.12 to 0.18)**	**20 (12–48)**	**4.6 (3.6 to 5.7)**
Ankle/foot	40	0.08 (0.06 to 0.11)	20 (12–48)	2.4 (1.8 to 3.3)
Knee	19	0.04 (0.02 to 0.06)	23 (13–44)	1.3 (0.8 to 2.0)
Hip/groin	8	0.02 (0.01 to 0.03)	20 (7–40)	0.5 (0.3 to 1.0)
Pelvis/sacrum	3	0.01 (0.00 to 0.02)	11 (10–14)	0.1 (0.0 to 0.2)
**Early YDP** *(U12–U14)*	**230**	**0.42 (0.37 to 0.48)**	**29 (17–61)**	**19.3 (16.9 to 21.9)**
Ankle/foot	48	0.09 (0.07 to 0.12)	27 (13–52)	4.3 (3.2 to 5.6)
Knee	85	0.16 (0.13 to 0.19)	27 (15–64)	6.4 (5.2 to 7.9)
Hip/groin	55	0.10 (0.08 to 0.13)	33 (21–50)	4.1 (3.1 to 5.3)
Pelvis/sacrum	19	0.03 (0.02 to 0.05)	20 (18–110)	2.2 (1.4 to 3.5)
**Late YDP** *(U15–U16)*	**112**	**0.38 (0.31 to 0.45)**	**38 (20–78)**	**20.3 (16.8 to 24.4)**
Ankle/foot	6	0.02 (0.01 to 0.05)	32 (28–40)	0.8 (0.3 to 1.7)
Knee	18	0.06 (0.04 to 0.10)	51 (27–91)	3.9 (2.5 to 6.2)
Hip/groin	57	0.19 (0.15 to 0.25)	34 (18–50)	8.9 (6.8 to 11.5)
Pelvis/sacrum	18	0.07 (0.04 to 0.11)	52 (19–83)	3.9 (2.5 to 6.2)
**PDP** *(U18 – U21)*	**37**	**0.10 (0.07 to 0.13)**	**35 (19–82)**	**4.6 (3.3 to 6.4)**
Ankle/foot	0	–	–	–
Knee	9	0.02 (0.01 to 0.04)	46 (27–77)	1.3 (0.7 to 2.6)
Hip/groin	24	0.06 (0.04 to 0.09)	34 (26–58)	2.7 (1.8 to 4.0)
Pelvis/sacrum	3	0.01 (0.00 to 0.02)	8 (7–68)	0.4 (0.1 to 1.1)

In some cases, injury location was not provided, therefore the totals per body location may not sum to the total number of injuries for that player phase.

FDP, Foundation Development Phase; PDP, Professional Development Phase; YDP, Youth Development Phase.

## Discussion

This prospective study explored apophyseal injuries in a cohort of 16 024 player-seasons among male academy football players. More than 10% of all musculoskeletal injuries in U11–U14 players were apophyseal injuries, with a cumulative prevalence ranging from 3.3 to 8.4% across these age groups. Recurrent apophyseal injuries accounted for 4.4% of cases, aligning closely with previous research (3.5%).^14^ Each academy experienced approximately 4–15 apophyseal injuries per season, and around one-quarter of these injuries became symptomatic outside of match or training contexts.

When compared with all musculoskeletal injuries sustained, apophyseal injuries accounted for the highest total days lost in the U9–U14 groups and ranked the third-highest total days lost in the U15–U16 group. The burden of growth-related injuries has previously been reported to be approximately twofold higher than muscle and ligament injuries in academy football,^30^ and in the present study, most apophyseal injuries were classified as severe, with a median recovery time exceeding 28 days. These data underscore the prevention and management of apophyseal injuries in an academy setting remains a priority. In addition, injury severity was higher in the U12–U16 groups compared with the U9–U11 group, suggesting a prolonged return-to-play period for older players. This delay in recovery could have significant long-term effects on player development and overall health. Apophyseal injuries can result in chronic pain, long-term disability, surgery or even cause players to abandon the sport.^11 31^ Injuries lasting more than 28 days can also hinder career progression in U17 and U19 elite youth footballers.^32^ Future research should explore the effectiveness of injury mitigation strategies in reducing the severity and burden of apophyseal injuries.^33^

Consistent with previous findings,^14^ the incidence and burden of apophyseal injuries were highest in U12–U16 groups, which coincides with the typical onset of the adolescent growth spurt in boys (around 11 years) and the typical age of peak height velocity (around 13.5 years).^34^ The incidence of ankle/foot, knee and hip/groin apophyseal injuries matches a prior study,^30^ and the higher burden of hip/groin and knee apophyseal injuries compared with the pelvis/sacrum and ankle/foot also aligns with previous literature.^14^ Thus, while ankle/foot and knee apophyseal injuries were the second most burdensome injuries in the U9–U11 and U12–U14 groups, respectively, it is knee and hip/groin apophyseal injuries that are of greater concern due to the higher burden. This is important, given that proximal apophyseal injuries typically occur at crucial ages in terms of academy progression, but might be mitigated through targeted interventions. A recent training intervention that adapted training load and content resulted in an 86% and 92% reduction in non-contact injury incidence and burden, respectively, in academy players at risk of growth-related injuries.^35^ These results suggest that training interventions may play a crucial role in mitigating the incidence and burden of growth-related injuries and warrant further exploration.

A clear distal-to-proximal gradient in the location of apophyseal injury was observed with player age. Injuries were more common in the ankle/foot in U9–U11 players, in the knee for U12 players and in the hip/groin for U15–U18 players. This pattern mirrors existing literature^14 19^ and likely reflects the higher risk of growth-related injuries in fast-growing players^30^ as well as the distal-to-proximal nature of the adolescent growth spurt.^36^ These findings highlight the need for developmentally tailored injury mitigation strategies to reduce the burden of apophyseal injuries in academy football players.

Interestingly, while the incidence of location-specific apophyseal injuries generally aligns with the period between initial appearance and closure of the calcaneal, tibial tubercle and iliac apophysis ossification centres in adolescent boys, and this pattern was not always reflected in the injury severity and burden. For example, although the tibial tubercle apophysis usually appears radiographically around age 11 in boys,^37^ the current study found a higher burden of knee apophyseal injuries in the U12–U14 and U15–U16 groups compared with the U9–U11 group. This likely results from both an increased incidence in the U12–U14 group and greater injury severity in the U15–U16 group (see table 2). Factors contributing to the higher burden in the U15–U16 group may include increased training load and technical demands with a more advanced category, and the prioritisation of short-term success over long-term player well-being, which can cause players to work through pain.^38^ In addition, greater physiotherapy awareness and the player’s maturational status are also likely to contribute to the higher injury burden, as players who are circa peak height velocity exhibit a high burden of growth-related injuries.^30^ Future research should incorporate growth and maturation status when investigating this topic.

This is the first study to report multiple apophyseal injuries in the U18–U21 group, with an injury incidence and burden similar to that observed in the U9–U11 group. While this is in contrast to previous literature reporting a significantly lower burden of growth-related injuries in U19 compared with U12 players,^20^ the larger sample size in the present study enabled the identification of more apophyseal injuries in older players. Specifically, ASIS apophyseal injuries accounted for the highest injury burden among all apophyseal injuries in the U18–U21 group. This suggests that apophyseal injuries continue to contribute to injury burden throughout the academy setting, supporting the need for mitigation strategies across all player phases. While muscle strains and ligament sprains are of greater concern in older players,^20^ investigation of proximal apophyseal injuries and mitigation strategies in the U18–U21 group may still be valuable.

### Clinical implications

This study offers valuable insights into the types, locations and burden of apophyseal injuries relative to player age, providing practitioners with a better understanding of the frequency of growth-related injuries and the typical return-to-play timelines across different player phases. The data presented can help shape specific injury mitigation strategies adopted in practice, which should be tailored to players’ age, maturity, and injury location, and applied across all developmental phases. Injury burden data highlight the need to prioritise prevention and management strategies for hip/groin and knee apophyseal injuries, particularly in U12–U16 players. Further investigation into the timing and causes of symptom onset would be valuable, given the number of apophyseal injuries that become symptomatic outside of match and training activities.

### Limitations

Of the musculoskeletal injuries recorded in the U9–U21 age groups, 501 (5.1%) ankle/foot injuries, 451 (4.6%) knee injuries and 416 (4.3%) hip/groin injuries were recorded using a generic OSICS code, without specifying the type of injury. This lack of detail may have led to an underestimation of the true incidence and burden of apophyseal injuries. The exposure data provided was aggregated across academies and player age groups rather than tracked at an individual level, thus the calculated incidence and burden of apophyseal injuries could be overestimated or underestimated. The approach to diagnosing apophyseal injuries can vary across academies, involving either imaging methods (e.g., radiography) or clinical reasoning by an experienced medical professional. For some apophyseal injuries, an estimated return date was used in the absence of an actual return date, which may have influenced the accuracy of severity and burden calculations. Cumulative prevalence was based on the number of apophyseal injuries, rather than the number of affected athletes, thus prevalence may be slightly overestimated. This study also did not consider individual growth rates or maturation levels, which are known to influence injury incidence, severity and burden.^8 19^ Finally, this study exclusively examined apophyseal injuries in male players. Future research should aim to consider these factors and explore apophyseal injuries in women academy players to provide a more comprehensive understanding of injury risk.

## Conclusion

This study reaffirms that apophyseal injuries are common in male academy football, with an incidence of approximately 0.4 injuries per 1000 hours in the U12–U16 age groups. While apophyseal injuries to the ankle/foot and knee were most common in U9–U14 groups, the severity of these injuries was higher in U12–U16 groups. Additionally, hip/groin and knee apophyseal injuries contributed to a higher injury burden across all player phases than ankle/foot and pelvis/sacrum apophyseal injuries. Interestingly, the overall burden of apophyseal injuries was similar between the U9–U11 and U18–U21 groups. The distal-to-proximal gradient in injury location with increasing player age emphasises the need for injury mitigation strategies that are tailored to specific ages, developmental phases and injury locations. Future research should further explore the effect of growth and maturation on injury burden, extend investigations to include growth-related injuries in female academy players and examine the timing and onset of symptoms.

## Data Availability

No data are available.
